# Air Pollution as a Risk Indicator for Periodontitis

**DOI:** 10.3390/biomedicines11020443

**Published:** 2023-02-02

**Authors:** Crystal Marruganti, Hye-Sun Shin, Seon-Ju Sim, Simone Grandini, Andreina Laforí, Mario Romandini

**Affiliations:** 1 Unit of Periodontology, Endodontology and Restorative Dentistry, Department of Medical Biotechnologies, University of Siena, 53100 Siena, Italy; 2 Department of Surgical, Medical and Molecular Pathology and Critical Care Medicine, University of Pisa, 56126 Pisa, Italy; 3 Sub-Unit of Periodontology, Halitosis and Periodontal Medicine, University Hospital of Pisa, 56124 Pisa, Italy; 4 Department of Dental Hygiene, Baekseok University, Cheonan 31065, Republic of Korea; 5 Big Data Statistics Institute, Cheonan 31065, Republic of Korea; 6 Division of Fixed Prosthodontics and Biomaterials, Clinic of Dental Medicine, University of Geneva, 1205 Geneva, Switzerland; 7 Faculty of Odontology, University Complutense, 28040 Madrid, Spain

**Keywords:** periodontal diseases, air pollution, airborne particulate matter, risk factors, epidemiology, KNHANES

## Abstract

Background: Air pollutants can influence local and systemic inflammation, oxidative stress and microbiome composition. Therefore, air pollution may potentially represent an unexplored modifiable risk indicator for periodontitis. The aim of the current cross-sectional study was to investigate the epidemiological association between outdoor air pollution and periodontitis in a representative sample of the South Korean population. Methods: A total of 42,020 individuals, which were representative of 35.2 million South Koreans, were examined. The mean annual levels of particulate matter of 10 μm (PM10), ozone, sulfur dioxide (SO_2_), nitrogen dioxide (NO_2_) and humidity, were studied. Periodontitis was defined according to the Community Periodontal Index (CPI ≥ 3). Simple and multiple regression analyses using four different models were applied. Results: Every 5-μg/m^3^ increase in PM10 (OR = 1.17; 95% confidence interval—CI: 1.11–1.24) and of 0.005 ppm in ozone levels (OR = 1.4; 95% CI: 1.00–1.30) were positively associated with periodontitis prevalence. Conversely, every 5% increase in humidity (OR = 0.94; 95% CI: 0.90–0.99) and 0.003 ppm increase in NO_2_ levels (OR = 0.93; 95% CI: 0.89–0.96) were inversely associated with periodontitis occurrence. Conclusions: In this nationally representative population several air pollutants were found to be associated with periodontitis occurrence. Hence, the present results suggest that air pollution may be a new modifiable risk indicator for periodontitis.

## 1. Introduction

According to the World Health Organization (WHO), air pollution is the largest environmental risk factor for human diseases, amounting to around one in eight deaths worldwide [1]. Pollutants of major public health concern include particulate matter (PM) (i.e., ash, smoke, etc.), as well as inorganic gases and secondary pollutants (i.e., sulfur dioxide-SO_2_, nitrogen dioxide-NO_2_ and ozone) generated due to chemical or photochemical reactions in the atmosphere. PM, together with SO_2_, NO_2_ and ozone, are co-pollutants and collectively constitute the major components of the Traffic-Related Air Pollution (TRAP) mixture [2].

From an epidemiological standpoint, exposure to air pollutants has been associated to an increased risk of developing several noncommunicable diseases (NCDs) and metabolic disorders, such as cardiovascular and respiratory diseases, inflammatory bowel diseases, obesity and type 2 diabetes mellitus (T2DM) [3]. Although there is a larger body of evidence regarding the adverse health effects of PM and ozone compared to SO_2_ and NO_2_, such air pollutants have been found to share the same causal pathways. In particular, the increased systemic inflammatory status and oxidative stress, as well as alterations in the microbiome composition of the gastrointestinal (GI) tract, have been demonstrated to collectively lend biological plausibility to the association between air pollution and such systemic diseases [4,5].

The mouth represents one of the main points of passage of air pollutants in the human body before their arrival in the lungs and the GI tract, with the consequent commencement of their systemic effects; hence, the direct contact of air pollutants with the periodontium takes place during their passage through the oral cavity. Therefore, due to its potential to influence both local and systemic inflammation, oxidative stress and microbiome composition, which constitute the main pathogenetic pathways involved in periodontal damage, air pollution may potentially represent an unexplored modifiable risk indicator for periodontitis.

However, despite its potential public health relevance, studies investigating the correlation between air pollution and periodontitis are lacking. Therefore, the aim of the current cross-sectional study was to investigate the epidemiological association between air pollution and periodontitis in a representative sample of the South Korean population.

## 2. Materials and Methods

This nationally representative cross-sectional study is reported according to the STrengthening the Reporting of OBservational studies in Epidemiology (STROBE) guidelines [6,7].

### 2.1. Study Sample and Survey Contents

The Korea National Health and Nutrition Examination Survey (KNHANES) is a nationwide cross-sectional epidemiologic survey conducted on an annually representative sample of the total non-institutionalized South Korean population [8]; it consists of three parts: the health interview, the health examination and the nutrition survey. Data for this study were obtained from the 2007–2009 sections of KNHANES IV, from the 2010 and 2012 sections of KNHANES V, and from the 2013–2015 sections of KNHANES VI. The 2011 section of KNHANES V was excluded as it did not pass quality control for periodontitis assessment. Further information regarding the study design and methods is reported elsewhere [8].

### 2.2. Sampling Methods and Inclusion Criteria

KNHANES employs a clustered, stratified, multistage sampling protocol with a rolling survey model.

A total of 192 Primary Sampling Units (PSUs), each consisting of around 50–60 households, were selected for each year from around 200,000 geographically defined areas of South Korea. A total of 20 targeted households were chosen through systematic sampling for each PSU, meaning that 3840 households were selected each year. All individuals living in the enrolled households who were at least one year old were included in the surveys. In order to represent the whole South Korean population, the sample participants were assigned sample weights, and the complex survey design, the null response and the post-stratification (performed by both gender and age) were encompassed.

### 2.3. Assessment of Study Variables

#### 2.3.1. Air Pollutants

The following air pollutants were considered for the present study: ambient PM with an aerodynamic diameter of less than 10 μm (PM10-measured in μg/m^3^ through β-ray absorption); ozone (measured in parts per million (ppm) through UV photometry); NO_2_ (measured in ppm through chemiluminescence); and SO_2_ (measured in ppm through UV fluorescence). Air pollutants levels were collected at 283 atmospheric monitoring stations in South Korea between 2007 and 2015, and the mean annual values of the 16 administrative divisions of South Korea (7 metropolitan cities and 9 provinces) were obtained from the Korea Ministry of Environment (http://me.go.kr), which was accessed on 28 July 2019, as previously reported [9].

Moreover, outdoor humidity (%) was considered as an additional air pollution factor, as it has been previously shown to influence the levels and effects of the other air pollutants [9,10]. Outdoor humidity levels were collected at 457 automatic weather stations between 2007 and 2015, and the mean annual values for the 16 administrative divisions of South Korea (7 metropolitan cities and 9 provinces) were obtained from the Korea Meteorological Administration website (https://data.kma.go.kr/), which was accessed on 29 July 2019.

The mean annual values from the monitoring site located in each KNHANES participant’s residential division in a specific year were used as proxies of exposure to ambient air pollutants and outdoor humidity levels for each specific participant (e.g., the average of the 2014 levels for each region were considered for KNHANES 2014 participants, etc.).

#### 2.3.2. Periodontitis

Periodontal assessments were carried out through the Community Periodontal Index (CPI) [11]. The measurements were performed through the walking probing method with a 0.5 mm ball-tipped CPI probe, and the examiners were calibrated to apply approximately a 20 g probing force.

The dentition was divided into six sextants represented by the following tooth numbers (FDI system): 18–14, 13–23, 24–28, 38–34, 33–43 and 44–48. A sextant was examined in case two or more teeth unscheduled for extraction were present. For the examination, ten index teeth were used: #17, #16, #11, #26, #27, #37, #36, #31, #46, #47 (FDI system). If a sextant missed one of the index teeth, an adjacent tooth was examined. In case no adjacent tooth was present, all the remaining teeth of that sextant were considered for examination.

The CPI ranged between 0 and 4, as follows: 0 (healthy), 1 (gingival bleeding after probing), 2 (calculus), 3 (probing pocket depth-PPD-between 3.5 and 5.5 mm) and 4 (PPD > 5.5 mm). Each sextant scored as high as its highest score.

For the current analysis, the periodontal status at the participant level was dichotomized as follows:

- Periodontitis case definition:
  - “No periodontitis” (CPI ≤ 2 in all sextants);
  - “Periodontitis” (CPI ≥ 3 in at least one sextant).
- Severe periodontitis (deep pocketing) case definition:
  - “No severe periodontitis” (CPI ≤ 3 in all sextants);
  - “Severe periodontitis” (CPI = 4 in at least one sextant).

#### 2.3.3. Covariates

The methods of the covariates assessment are reported in Supplementary Materials (S1).

### 2.4. Statistical Analyses

The statistical plan was similar to the one that was previously reported [12,13,14]. Briefly, analyses for complex samples’ survey data with a design-based approach [15] were performed in order to generalize the results to the entire South Korean population. All missing data with any assumption were handled with complete case analyses with covariates adjustment, consistently with the current statistical literature orientation [16] (Table S1). Descriptive characteristics were summarized for the whole population and by periodontal status. Categorical data were reported as number (percentage—%), while continuous variables were reported as mean (relative standard deviation—RSD).

Simple and multiple logistic regression analyses were then applied in order to examine the association between air pollutants levels and periodontitis, adjusting or not (“crude”) for potential confounders (selected according to external knowledge). The following models were used:

a Crude;
b Model 1 (age + gender);
c Model 2 (Model 1 + smoking status + educational level + income);
d Model 3 (Model 2 + area of residence + marital status + BMI + AUDIT score + hypertension);
e Model 4 (Model 3 + frequency of toothbrushing + use of interproximal toothbrush + stress).

Model 2 was taken as the reference model, since Models 3 and 4 were also adjusted for factors potentially involved in the causal pathway between air pollution and periodontitis (e.g., BMI and hypertension). The odds ratios (ORs) (95% confidence intervals—CIs) obtained from the multiple regression analyses were reported, as well as the *p*-values derived from an F-test.

All analyses were performed through ad hoc statistical software (SPSS version 25, IBM Corp, Armonk, NY, USA), setting the level of significance at 5% and reporting two-tailed *p*-values.

## 3. Results

The sampling strategy led to the selection of 82,031 people, out of whom 64,835 (77.4%) agreed to participate. Of those participants, 61,475 (74.9%) took part in the health interview and examination. The present analysis included only those who received the periodontal examination, for a total of 42,020 participants, which were representative of 35.2 million individuals (Figure S1).

### 3.1. Descriptive Statistics

Table 1 shows the descriptive statistics of the study participants overall and by periodontal status. The weighted mean age was 44.91 (0.003) years; the majority of participants were women (weighted 50.4%), non-smokers (weighted 68.4%) and living in an urban area (weighted 82.0%).

A total of 13,246 participants had periodontitis defined as CPI ≥ 3 (weighted n. 10,024,655—weighted 18.5%), while 3205 participants had severe periodontitis defined as CPI = 4 (weighted n. 2,482,456—weighted 7.0%).

As for air pollution factors, the overall mean levels of humidity, PM10, ozone, NO_2_ and SO_2_ were 65.940 (0.001) %, 50.011 (0.002) μg/m^3^, 0.024 (0.002) ppm, 0.025 (0.03) ppm and 0.005 (0.004) ppm, respectively.

### 3.2. Air Pollution and Periodontitis

Table 2 shows the ORs (95% CI) for the association between air pollutants and periodontitis (CPI ≥ 3 case definition). Taking Model 2 as a reference, every 5% increase in humidity levels showed an inverse and statistically significant association to periodontitis (OR = 0.94, 95% CI: 0.90–0.99); the results were consistent in Models 3 and 4.

Moreover, while every 5 μg/m^3^ increase in PM10 significantly increased the odds for periodontitis in Model 2 (OR = 1.17; 95% CI: 1.11–1.24), every 0.003 ppm increase in NO2 resulted to be inversely related (OR = 0.93; 95% CI: 0.89–0.96); the results were also consistent in Models 3 and 4.

Every 0.005 ppm increase in ozone levels was significantly associated with periodontitis in Model 2 (OR = 1.14; 95% CI: 1.00–1.30), consistently with Models 3 and 4 (although in Model 4 it was just below the statistical significance). 

Moreover, every 0.005 ppm increase in SO_2_ levels was significantly associated with periodontitis in Model 2 (OR = 1.28; 95% CI: 1.00–1.65), consistently with Models 3 and 4 (although neither reached statistical significance).

### 3.3. Air Pollution and Severe Periodontitis

Table 3 reports the ORs (95% CI) for the association between air pollutants and severe periodontitis (CPI = 4 case definition). Humidity, NO_2_ and SO_2_ were not significantly associated with severe periodontitis in any of the models considered.

Severe periodontitis was associated to every 5 μg/m^3^ increase in PM10 and every 0.005 ppm increase in ozone levels in Model 2 (OR = 1.08; 95% CI: 1.00–1.16 and OR = 1.25; 95% CI: 1.01–1.51, respectively); the results for PM10 were consistent in Models 3 and 4 (albeit not significantly), whereas the results for ozone were consistent in Model 4 but not in Model 3.

## 4. Discussion

In the current study, every minor increase in outdoor PM_10_, ozone and SO_2_ levels resulted to be independently associated with periodontitis. Conversely, NO_2_ and humidity levels were inversely correlated with periodontitis occurrence. When focusing on severe periodontitis, the results were consistent for PM_10_ and ozone levels, while the relationship disappeared with SO_2_, NO_2_ and humidity.

Air pollutants can mainly enter the human body through two mechanisms: ingestion and inhalation. While there is plenty of evidence regarding the pathways and mechanisms that lead to both the inhalation and ingestion of air pollutants to trigger the systemic effects stemming from the lungs and the GI tract [17], little information is available regarding the “direct” local effects of air pollutants on the oral cavity. However, pollutants can come into direct contact with the periodontium in various ways. First, inhalation can take place not only through the nose, but also through the mouth, hence causing a passage of moistened but unfiltered air through the mouth; this passage can also occur during exhalation. Secondly, air pollutants come into direct contact with the periodontium via the ingestion of contaminated food and water [18,19]. Hence, a continuous direct interaction between air pollutants and the periodontal tissues takes place in the oral cavity.

From a pathogenetic standpoint, although each pollutant retains its own peculiarities, three main pathways are likely to be recognized as possible mechanisms of association with periodontitis: inflammation, oxidative stress and microbiome alteration. Such mechanisms can influence periodontal health through both a “direct” and an “indirect” effect. While the “direct” effect indicates the local action exerted by air pollutants on the oral cavity, the “indirect” effect encompasses the impact of air pollutants on periodontal health as a result of their repercussions on the systemic level [20]. Therefore, it can be hypothesized that a multiplier effect takes place between the “direct” and “indirect” effects exerted by air pollutants on the periodontium, ultimately and collectively leading to the significant association between air pollution and periodontitis observed in the current study.

From the available literature, it can be hypothesized that the direct contact of air pollutants with the periodontal tissues may lead to increased local inflammation and oxidative stress, similarly to what happens in the lungs. In fact, after inhalation, air pollutant particles, especially PM_10_, can deposit in the lungs and reach the upper bronchi, thus activating an inflammatory response in the alveolar macrophages (AM), which is characterized by a potent cytokine cascade and increased production of reaction oxidant species (ROS) [21]; hence, the inhalation of air pollutants stimulates pulmonary inflammation [22]. Similarly, the deposition of air pollutant particles and their components (i.e., combustion particles and transition metals, such as iron, copper and zinc) on the oral mucosa and the periodontium may stimulate the same inflammatory pathways and oxidative stress in the oral cavity, thus mimicking the process triggered in the lungs. Therefore, such a “direct” local effect, which is characterized by the increased cytokine expression and ROS concentration in the periodontium, could induce periodontal inflammation, and thus, contribute to the development of periodontitis. Nonetheless, evidence is needed to investigate the precise molecular pathways involved in the “direct” local effect of air pollutants deposition on the periodontium.

On the other hand, the impact of air pollutants inhalation or ingestion on systemic health has been investigated by many studies [23,24], and this constitutes the “indirect” effect of air pollutants exposure on periodontal health. In particular, the cytokines and ROS produced in the lungs spread into the circulation, thus stimulating the liver to produce acute phase proteins, such as C-reactive protein (CRP) and fibrinogen [17,25]. In turn, the induced systemic proinflammatory state [26,27] could affect the periodontium by also augmenting cytokine expression in the periodontal tissues, increasing vascular permeability and stimulating bone resorption by enhancing the NF-κB pathway [20,28]. Specifically, previous studies have shown the pro-inflammatory effects of SO_2_ exposure in both preclinical and clinical models [29]. In keeping with this, airborne PM, as well as ozone, elicit the production of pro-inflammatory mediators via the oxidative stress pathway and via the elicitation of an inflammatory response in macrophages [30,31]. However, the inverse association found between periodontitis and NO2 is not fully understood, since this air pollutant has also previously been shown to be linearly related to a pro-inflammatory status [32].

Recent, substantial evidence exists regarding the effects of pollutants exposure on the relative changes in the microbiota function and composition of the GI tract [33,34]. The direct contact between air pollutants and the GI tract may indeed induce changes in the microbiota composition from its proximal to its most distal parts [24,35]. Consequently, given that the oral cavity represents the first point of entry to the GI tract, it is plausible to assume that the deposition of air pollutants on the periodontium and the oral mucosa induces significant changes in the oral microbiome composition.

The adverse effects of air pollution on general health were previously found to be influenced by humidity levels. While, on one hand, high outdoor humidity increases the time that air pollutants stay airborne, on the other hand, it can increase their mass and size through water absorption [36]. Therefore, since the adverse health effects of air pollutants (especially PM) increase with a decreasing size of its particles, humidity may mitigate their impact. The present findings also indirectly support this attenuating effect of humidity on air pollutants for periodontitis.

To the best of the authors knowledge, no study has previously investigated the epidemiological association between air pollution and periodontitis. However, a panel study monitored the levels of circulating hS-CRP in 100 periodontitis patients and 100 periodontally healthy adults over a period of 2 years [37]. A 10 μg/m^3^ increase in PM_2.5_ was associated with a significantly higher hs-CRP increase in periodontitis patients compared to periodontally healthy adults (9.62% vs. 1.17%). Therefore, the results obtained by Yang and coworkers (2015) may lead to the conclusion that patients affected by periodontitis are more susceptible than healthy individuals to the systemic effects caused by PM exposure. Moreover, both air pollution and periodontitis have been found to be independently associated with several diseases and conditions, such as cardiovascular and respiratory diseases [2,38], obesity and T2DM [5], inflammatory bowel diseases [39], COVID-19 [40,41], cancer [42,43], cognitive impairment [44], biological ageing [45] and even mortality [46,47,48]. Consequently, periodontitis may potentially represent a mediator in the causal pathway relating air pollution with those systemic diseases and conditions.

Although analyses were conducted using both CPI ≥ 3 and CPI = 4 case definitions, the use of the CPI for periodontitis assessments may result in information bias; future studies should preferably use the 2018 classification system [49,50]. Information bias may also exist in relation to air pollution, since mean annual values in the participants’ residential divisions may not have accurately reflected true exposure to air pollutants. Other limitations worth mentioning are the lack of data regarding fine PM (PM_2.5_), which prevented its analysis as a possible additional risk indicator; the risk of residual confounding; and the cross-sectional design, which does not allow to verify the temporality of such associations. Finally, even if representative of the whole of South Korea, the present findings may potentially be unapplicable to other populations.

## 5. Conclusions

Exposure to air pollutants was associated with periodontitis, even after adjustments for confounders. The identification of air pollution as a modifiable risk indicator for periodontitis could lay the groundwork for the implementation of new environmental policies that could reduce the burden of periodontitis, as well as other NCDs, on global public healthcare [51].

## Figures and Tables

**Table 1:** Characteristics of the study population and air pollution factors, overall and by periodontal status.

		CPI ≥ 3		CPI = 4	
Variables	Overall ^a^	No Periodontitis ^b^	Periodontitis ^b^	No Severe Periodontitis ^c^	Severe Periodontitis ^c^
Age (years), mean (RSD)	44.91 (0.003)	41.71 (0.004)	52.97 (0.004)	44.16 (0.003)	54.88 (0.006)
Gender, N (%)					
Men	17,876 (49.6)	11,055 (46.1)	6821 (58.3)	16,023 (48.6)	1853 (62.7)
Women	24,144 (50.4)	17,719 (53.9)	6425 (41.7)	22,742 (51.4)	1402 (37.3)
Smoking status, N (%)					
Non-smokers	29,532 (68.4)	21,193 (71.5)	8339 (60.3)	27,539 (69.1)	1993 (58.8)
Current smokers	11,336 (31.6)	6835 (28.5)	4501 (39.7)	10,187 (30.9)	1149 (41.2)
Educational level, N (%)					
Primary school	9974 (17.3)	5442 (13.1)	4532 (28.1)	8881 (16.5)	1093 (29.1)
Middle school	4425 (9.7)	2542 (7.8)	1883 (14.4)	3917 (9.2)	508 (16.1)
High school	13,999 (39.5)	10,221 (41.7)	3778 (34.0)	13,083 (40.0)	916 (33.5)
University/College	12,061 (33.5)	9548 (37.4)	2513 (23.5)	11,473 (34.4)	588 (21.3)
Monthly household income, N (%)					
Low	7945 (15.3)	4623 (13.1)	3322 (20.8)	7126 (14.8)	819 (21.6)
Middle-low	10,449 (25.3)	6957 (24.7)	3492 (26.8)	9592 (25.2)	857 (26.4)
Middle-high	11,330 (29.4)	8079 (30.1)	3251 (27.4)	10,527 (29.6)	803 (26.5)
High	11,658 (30.1)	8716 (32.0)	2942 (25.1)	10,934 (30.4)	724 (25.4)
Area of residence, N (%)					
Urban	32,902 (82.0)	23,302 (84.2)	9600 (76.3)	30,597 (82.6)	2305 (73.1)
Rural	9118 (18.0)	5472 (15.8)	3646 (23.7)	8168 (17.4)	950 (26.9)
Marital status, N (%)					
Never married	6196 (22.2)	5578 (28.0)	618 (7.5)	6107 (23.5)	89 (4.2)
Married and living with a spouse	30,180 (67.6)	19,854 (63.5)	10,326 (78.0)	27,588 (66.6)	2592 (81.0)
Married but living alone	5504 (10.2)	3252 (8.5)	2252 (14.5)	4947 (9.9)	557 (14.8)
BMI (kg/m²), mean (RSD)	23.71 (0.001)	23.49 (0.001)	24.25 (0.002)	23.65 (0.001)	24.42 (0.003)
Alcoholism (AUDIT score), mean (RSD)	6.95 (0.007)	6.72 (0.009)	7.55 (0.013)	6.87 (0.008)	8.04 (0.025)
Hypertension status, N (%)					
Normal	18,560 (49.9)	14,342 (55.4)	4218 (36.0)	17,644 (51.2)	916 (32.3)
Pre-hypertension	9709 (24.7)	6513 (24.3)	3196 (26.0)	8897 (10872)	812 (26.5)
Hypertension	12,255 (25.4)	6934 (20.3)	5321 (38.0)	10,872 (24.2)	1383 (41.2)
Tooth-brushing frequency, N (%)					
0–1 per day	6120 (13.9)	3612 (12.2)	2508 (18.2)	5458 (13.4)	662 (19.7)
2 per day	16,760 (39.0)	11,051 (37.6)	5709 (42.5)	15,379 (38.8)	1381 (41.7)
≥3 per day	19,063 (47.1)	14,066 (50.2)	4997 (39.2)	17,856 (47.7)	1207 (38.5)
Use of interproximal toothbrush, N (%)					
No	30,383 (82.4)	20,544 (81.7)	9839 (84.0)	28,000 (82.3)	2383 (84.2)
Yes	6216 (17.6)	4517 (18.3)	1699 (16.0)	5791 (17.7)	425 (15.8)
Stress, N (%)					
Not/slightly stressed	29,920 (72.1)	20,185 (71.0)	9735 (75.1)	27,483 (71.8)	2437 (77.0)
Moderately/highly stressed	10,914 (27.9)	7827 (29.0)	3087 (24.9)	10,214 (28.2)	700 (23.0)
Air pollution factors					
Humidity (%), mean (RSD)	65.940 (0.001)	65.861 (0.001)	66.136 (0.001)	65.910 (0.001)	66.335 (0.002)
PM_10_ (μg/m^3^), mean (RSD)	50.011 (0.002)	49.917 (0.002)	50.248 (0.003)	50.032 (0.002)	49.736 (0.004)
Ozone (ppm), mean (RSD)	0.024 (0.002)	0.024 (0.003)	0.025 (0.003)	0.024 (0.002)	0.025 (0.005)
Nitrogen dioxide (ppm), mean (RSD)	0.025 (0.003)	0.026 (0.004)	0.025 (0.005)	0.026 (0.003)	0.024 (0.008)
Sulfur dioxide (ppm), mean (RSD)	0.005 (0.004)	0.005 (0.005)	0.005 (0.005)	0.005 (0.004)	0.005 (0.007)

CPI: Community Periodontal Index; RSD: relative standard deviation; BMI: body mass index; AUDIT: Alcohol Use Disorders Identification Test score; PM_10_: particulate matter. Data are presented as unweighted N (weighted %) for binary and categorical variables and as weighted mean (weighted RSD) for continuous variables. N is the unweighted population size for each group; the sum of N varied according to missing data for each variable. ^a^ Overall: N(unweighted) = 42,020; n(weighted) = 35,224,590. ^b^ CPI ≥ 3 case definition. No periodontitis: N(unweighted) = 28,774; n(weighted) = 25,199,934 (71.5%). Periodontitis: N(unweighted) = 13,246; n(weighted) = 10,024,655 (18.5%). ^c^ CPI = 4 case definition. No Severe Periodontitis: N(unweighted) = 38,765; n(weighted) = 32,742,134 (93.0%). Severe Periodontitis: N(unweighted) = 3255; n(weighted) = 2,482,456 (7.0%).

**Table 2:** Crude and adjusted ORs (95% CI) for the association between periodontitis (CPI ≥ 3) and multiple air pollution factors.

Variables	CPI ≥ 3 Periodontitis Case Definition				
	Crude	Model 1	Model 2	Model 3	Model 4
Humidity, 5% increase	0.95 (0.91/1.00)	0.94 (0.89/0.99) *	0.94 (0.90/0.99) *	0.92 (0.87/0.97) *	0.92 (0.86/0.97) *
PM_10_, 5 μg/m^3^ increase	1.17 (1.11/1.23) *	1.20 (1.14/1.27) *	1.17 (1.11/1.24) *	1.18 (1.11/1.26) *	1.19 (1.12/1.27) *
Ozone, 0.005 ppm increase	1.14 (1.01/1.29) *	1.09 (0.96/1.24)	1.14 (1.00/1.30) *	1.18 (1.11/1.26) *	1.16 (0.98/1.37)
Nitrogen dioxide, 0.003 ppm increase	0.91 (0.88/0.94) *	0.91 (0.87/0.94) *	0.93 (0.89/0.96) *	0.92 (0.88/0.96) *	0.92 (0.87/0.96) *
Sulfur dioxide, 0.005 ppm increase	1.37 (1.08/1.74) *	1.39 (1.10/1.77) *	1.28 (1.00/1.65) *	1.29 (0.96/1.73)	1.29 (0.96/1.73)

OR: odds ratio; CI: confidence interval; CPI: Community Periodontal Index; PM: particulate matter; ppm: parts per million. Model 1: adjusted for age and gender. Model 2: Model 1 + adjustment for smoking, educational level and income. Model 3: Model 2 + adjustment for area of residence, marital status, BMI, AUDIT score and hypertension. Model 4: Model 3 + adjustment for frequency of toothbrushing, use of interproximal toothbrush and stress. * *p* < 0.05.

**Table 3:** Crude and adjusted ORs (95% CI) for the association between periodontitis (CPI = 4) and multiple air pollution factors.

Variables	CPI = 4 Periodontitis Case Definition				
	Crude	Model 1	Model 2	Model 3	Model 4
Humidity, 5% increase	1.01 (0.95/1.08)	0.99 (0.93/1.06)	0.99 (0.93/1.07)	1.01 (0.93/1.10)	1.01 (0.93/1.10)
PM_10_, 5 μg/m^3^ increase	1.10 (1.02/1.17) *	1.11 (1.04/1.19) *	1.08 (1.00/1.16) *	1.04 (0.96/1.13)	1.06 (0.97/1.15)
Ozone, 0.005 ppm increase	1.30 (1.09/1.56) *	1.27 (1.06/1.53) *	1.25 (1.04/1.51) *	1.12 (0.90/1.41)	1.32 (1.07/1.64) *
Nitrogen dioxide, 0.003 ppm increase	0.93 (0.88/0.99)	0.95 (0.89/1.00)	0.95 (0.90/1.01)	0.95 (0.89/1.02)	0.98 (0.91/1.05)
Sulfur dioxide, 0.005 ppm increase	1.18 (0.88/1.59)	1.14 (0.85/1.53)	1.06 (0.78/1.45)	1.07 (0.74/1.54)	1.17 (0.80/1.69)

OR: odds ratio; CI: confidence interval; CPI: Community Periodontal Index; PM: particulate matter; ppm: parts per million. Model 1: adjusted for age and gender. Model 2: Model 1 + adjustment for smoking, educational level and income. Model 3: Model 2 + adjustment for area of residence, marital status, BMI, AUDIT score and hypertension. Model 4: Model 3 + adjustment for frequency of toothbrushing, use of interproximal toothbrush and stress. * *p* < 0.05.

## Data Availability

The data used for this study are openly available to the public on request from the KNHANES website (https://knhanes.cdc.go.kr/knhanes/eng/index.do; accessed on 1 July 2019).

## Supplementary Materials

The following supporting information can be downloaded at: https://www.mdpi.com/article/10.3390/biomedicines11020443/s1, Supplementary Material. Covariate assessment methods; Table S1. Amount of missing data in the study population (n = 42,020) for each variable considered; Figure S1: flow diagram showing the participant selection process.
