# Supplementary material for: Air Pollution as a Risk Indicator for Periodontitis
Source: Biomedicines. 2023 Feb 2;11(2):443. doi: 10.3390/biomedicines11020443 (PMC9953183; doi:10.3390/biomedicines11020443)

Supplementary Appendix. Covariate assessment methods.

As socio-demographic characteristics, the following variables were considered: age, educational level, marital status and monthly household income. The educational level was evaluated as the highest diploma obtained (primary school, middle school, high school and university/college). The marital status was categorized in 3 groups: never married, married and living with a spouse, married but living alone (due to divorce or the death of a spouse). Income was assessed as the family income adjusted for the number of family members; on this basis, participants were classified, in the initial sample, into quartiles groups (low, middle low, middle high and high).

As systemic health variables, Body Mass Index (BMI) and hypertension status were considered. All of them were measured during the health examination. Body height and weight were respectively measured to the nearest 0.1 cm and 0.1 kg, with the participants in light indoor clothing without shoes. BMI was then calculated (kg/m^2). Hypertension status was categorized in normotension, prehypertension and hypertension. Normotension was defined as having both systolic blood pressure (SBP)<120 and diastolic blood pressure (DBP)<80, prehypertension was defined as having either $120 \leq SBP < 140$ mmHg or $80 \leq DBP < 90$ mmHg, while hypertension was defined as having an average $SBP \geq 140$mmHg or $DBP \geq 90$mmHg or being medicated for hypertension.

As general health behaviors, the following self-reported variables were considered: smoking status, alcohol consumption and stress. Regarding smoking status, the subjects were divided into two groups: non-smokers (including former smokers) and current smokers. Alcohol consumption was assessed with the AUDIT score (Alcohol Use Disorders Identification Test), which was considered as a continuous variable. Self-reported stress was categorized as no/slightly stressed and moderately/highly stressed.

As self-reported oral health behavior, the toothbrushing frequency per day (0-1 time/day, 2 times/day, ≥3 times/day) was considered.

Table S1. Amount of missing data in the study population (n=42,020) for each variable considered.

Variables	Collected data, N (%)	Missing data, N (%)
**Periodontitis**	42,020 (100.0)	0 (0.0)
**Humidity**	42,020 (100.0)	0 (0.0)
**PM10**	42,020 (100.0)	0 (0.0)
**Ozone**	42,020 (100.0)	0 (0.0)
**Nitrogen dioxide**	42,020 (100.0)	0 (0.0)
**Sulfur dioxide**	42,020 (100.0)	0 (0.0)
**Age**	42,020 (100.0)	0 (0.0)
**Gender**	42,020 (100.0)	0 (0.0)
**Smoking Status**	40,868 (97.3)	1,152 (2.7)
**Educational Level**	40,459 (96.3)	1,561 (3.7)
**Monthly household income**	41,382 (98.5)	638 (1.5)
**Region of residence**	42,020 (100.0)	0 (0.0)
**Marital status**	41,880 (99.7)	140 (0.3)
**BMI**	41,921 (99.8)	99 (0.2)
**Alcoholism**	31,219 (74.3)	1,0801 (25.7)
**Hypertension Status**	40,524 (96.4)	1,496 (3.6)
**Tooth brushing frequency**	41,943 (99.8)	77 (0.2)
**Use of interproximal toothbrush**	36, 599 (87.1)	5,421 (12.9)
**Stress**	40, 834 (97.2)	1,186 (2.8)

Figure S1: flow diagram showing the participants selection process.

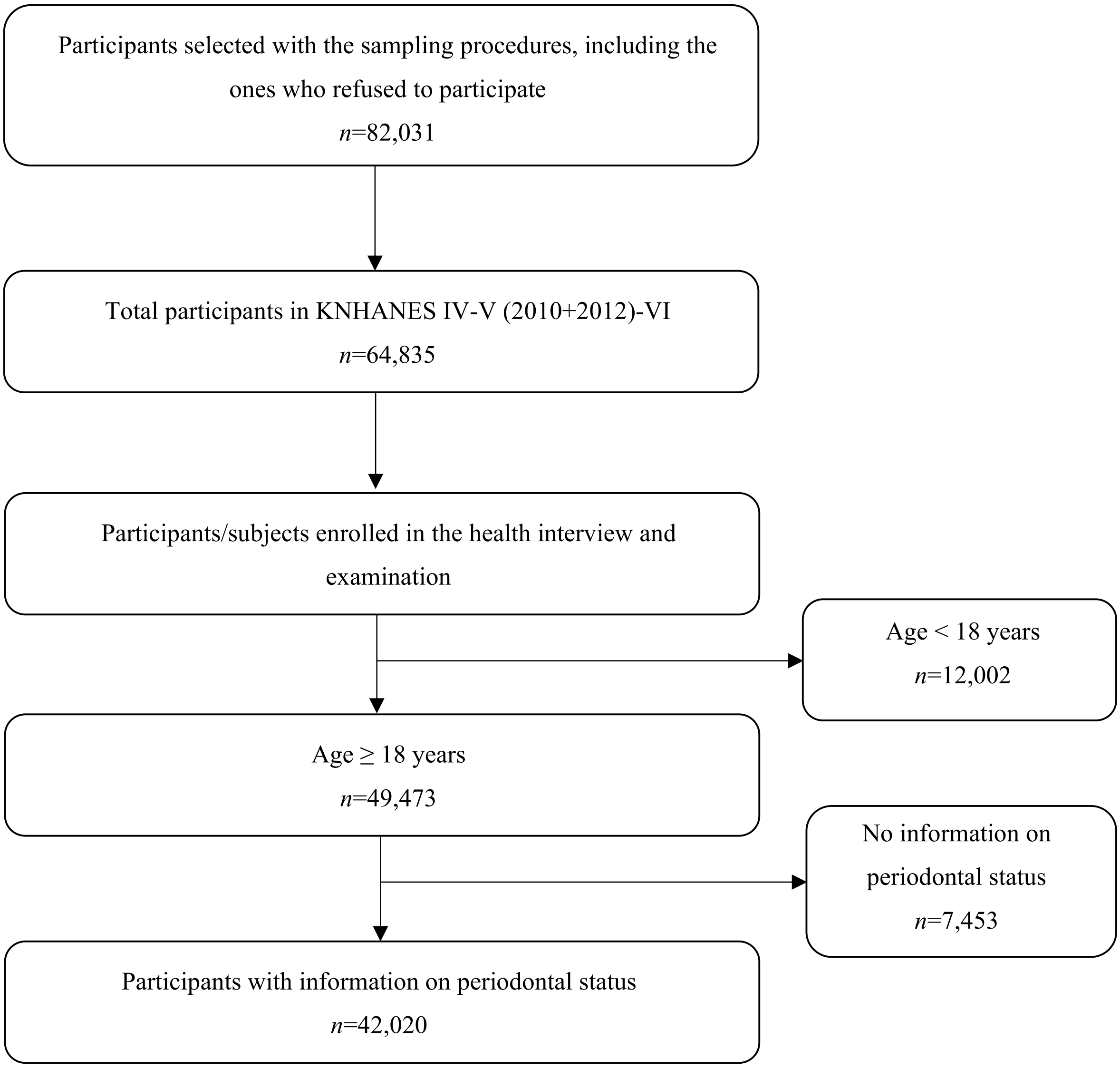

Supplement: Supplementary file 1 [file biomedicines-11-00443-s001.zip › biomedicines-2172406-supplementary.pdf]
